# The relationship between serum 25-hydroxyvitamin D and parathyroid hormone concentration in assessing vitamin D deficiency in pet rabbits

**DOI:** 10.1186/s12917-020-02599-7

**Published:** 2020-10-27

**Authors:** J. Mäkitaipale, S. Sankari, H. Sievänen, O. Laitinen-Vapaavuori

**Affiliations:** 1 Department of Equine and Small Animal Medicine, Faculty of Veterinary Medicine, University of Helsinki, P.O. Box 57, 00014 Helsinki, Finland; 2 The UKK Institute for Health Promotion Research, Kaupinpuistonkatu 1, 33500 Tampere, Finland

**Keywords:** Rabbit, Vitamin D, 25-hydroxyvitamin D, Parathyroid hormone, Calcium, Phosphorus, Bone density, Vitamin D deficiency, Metabolic bone disease

## Abstract

**Background:**

Vitamin D deficiency and related metabolic bone diseases in pet rabbits have been intermittently debated. In human research, the parathyroid hormone concentration in relation to the 25-hydroxyvitamin D concentration is used to determine vitamin D deficiency. Thus, this study aimed to identify the breakpoint in the 25-hydroxyvitamin D concentration indicating a significant change in the parathyroid hormone concentration in 139 pet rabbits. An enzyme immunoassay kit was used for 25-hydroxyvitamin D analysis and the intact parathyroid hormone (PTH 1–84) immunoradiometric assay kit for parathyroid hormone analysis. The mid-tibial cortical bone density was measured using peripheral quantitative computed tomography. A segmented linear regression analysis was performed, with the 25-hydroxyvitamin D concentration as the independent variable, and parathyroid hormone, ionised calcium, total calcium, inorganic phosphorus concentrations and the mid-tibial cortical density as the dependent variables.

**Results:**

The breakpoint for the parathyroid hormone concentration occurred at a 25(OH)D concentration of 17 ng/mL, whereas the cortical bone density breakpoint occurred at a 25-hydroxyvitamin D concentration of 19 ng/mL. No breakpoints were found for ionised calcium, total calcium or phosphorus.

**Conclusions:**

These results suggest that a serum 25-hydroxyvitamin D concentration of 17 ng/mL serves as the threshold for vitamin D deficiency in rabbits. Nearly one-third of the rabbits had a serum 25-hydroxyvitamin D concentration below this threshold. Concerns persist regarding the high prevalence of vitamin D deficiency in pet rabbits and the possible health consequences caused by a chronic vitamin D deficiency, including the risk for metabolic bone diseases.

## Background

Vitamin D deficiency and the existence of related metabolic bone diseases in pet rabbits are topics of intermittent debate [1–5]. The question regarding the existence of metabolic bone diseases arose 20 years ago following a study regarding the aetiology of dental disease, whereby dietary hypocalcaemia and hypovitaminosis D were suspected as causal factors [6]. Subsequently, findings that hutch rabbits with dental disease had a higher parathyroid hormone (PTH) and a lower total calcium (Ca) concentration compared to healthy rabbits in free-range conditions advanced that theory [7].

Maintaining a skeletal calcium balance is the most important function of vitamin D, along with the important role played in many other metabolic functions as well. Vitamin D deficiency is associated with an elevated PTH concentration, which increases calcium reabsorption and phosphorus excretion in the renal tubulus, and the conversion of 25-hydroxyvitamin D (25(OH)D) to active 1.25-dihydroxyvitamin D in the kidneys. In addition, 1.25-dihydroxyvitamin D increases active calcium absorption in the intestines and stimulates osteoclastic bone resorption, releasing calcium from the bone to serum. In human research, PTH concentrations in relation to the 25(OH)D concentration commonly serve to evaluate vitamin D deficiency levels [8–10]. Because calcium absorption in the intestines is vitamin D-dependent in humans and many other mammals, vitamin D deficiency appears to reduce the serum calcium concentration leading to increased PTH concentrations. Furthermore, the 25(OH)D concentration, where the PTH concentration begins to increase or no longer decreases, may mark vitamin D deficiency [11]. Calcium absorption is primarily passive in rabbits, although higher PTH concentrations occur more frequently in vitamin D deficient rabbits than in control rabbits [12, 13].

Twenty years after the first studies proposing vitamin D deficiency as a common problem in pet rabbits, studies focusing on vitamin D deficiency and possible vitamin D deficient levels remain lacking. This study, therefore, aimed to determine the breakpoint in 25-hydroxyvitamin D concentration linked to changes in the parathyroid hormone concentration in pet rabbits. We also studied whether similar breakpoints exist when the 25(OH)D concentration is compared to several bone biomarkers and the bone density. Significant breakpoints may indicate disturbances in calcium and bone metabolism and provide a clinically useful threshold value to evaluate the risk for metabolic bone disease.

## Results

Table 1 summarises the clinical and biochemical characteristics of the rabbits. The mean 25-hydroxyvitamin D concentration was 25.9 ng/mL and the mean serum parathyroid hormone concentration was 14.8 pg/mL. PTH concentration was below limit of detection in 8 samples. Table 2 shows the univariate correlations between biochemical characteristics and rabbit’s age and weight. The 25(OH)D concentration correlated significantly with PTH (*r* = -0.19, *P* = 0.028) and with body weight (*r* = 0.20, *P* = 0.022). PTH concentration correlated significantly with ionised calcium (iCa) (*r* = -0.26, *P* = 0.008). Cortical bone density correlated significantly with iCa (*r* = 0.46, *P* < 0.001), inorganic phosphorus (P) (*r* = -0.30, *P* = 0.009), age (*r* = 0.32, *P* = 0.007), and weight (*r* = 0.32, *P* = 0.006). Total calcium correlated significantly with ionised calcium (*r* = 0.60, *P* < 0.001). Inorganic phosphorus correlated significantly with age (*r* = -0.37, *P* < 0.001).

**Table 1:** Descriptive data on the age, weight, serum 25-hydroxyvitamin D, parathyroid hormone, total calcium, inorganic phosphorus, ionised calcium and mid-tibial cortical bone density of pet rabbits

Parameter	n^a^	Mean	SD	Range
Age (years)	134	2.7	2.0	0.1–9.3
Weight (kg)	133	2.4	1.2	0.3–6.2
25-hydroxyvitamin D (ng/mL)	139	25.9	11.7	4.5–67.5
Parathyroid hormone (pg/mL)	139^b^	4.8	7.6	< 0.5–49.1
Total calcium (mmol/L)	134	3.65	0.19	2.9–4.0
Ionised calcium (mmol/L)	104	1.64	0.12	1.2–1.9
Inorganic phosphorus (mmol/L)	137	1.23	0.19	0.6–2.5
Mid-tibial cortical bone density (mg/cm^3^)	73	1392.5	81.4	926.2–1498.3

^a^Number of rabbits

^b^PTH concentration below limit of detection (LOD) in 8 samples (mean 0.26, range 0.04—0, 4 pg/mL)

**Table 2:** Correlations between 25-hydroxyvitamin D (25(OH)D), parathyroid hormone (PTH), ionised calcium (iCa), total calcium (Ca), and inorganic phosphorus (P) concentrations, cortical bone density (CortD), age and body weight

Variable	PTH	CortD	Ca	iCa	P	Age	Weight
25(OH)D	-0.19*	0.17	0.05	-0,01	0.05	-0.10	0.20*
PTH	-	-0.20	-0.16	-0.26**	0.14	-0.02	-0.11
CortBD	-	-	0.15	0.46***	-0.30**	0.32**	0.32**
Ca	-	-	-	0.60***	-0.10	0.11	-0.06
iCa	-	-	-	-	-0.19	0.08	0.05
P	-	-	-	-	-	-0.37***	-0.12

*p < 0.05, **p < 0.01, ***p < 0.001

**Fig. 1:**
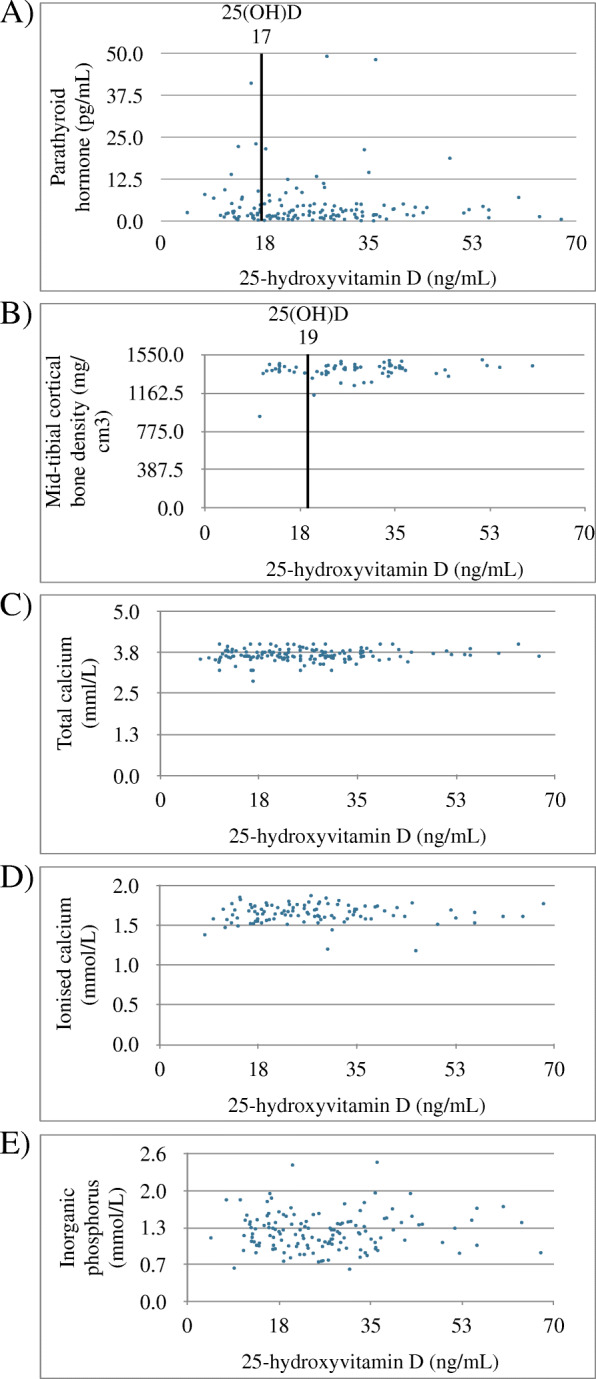
Scatterplots showing the relationships between the (**a**) serum parathyroid hormone (PTH), **b** the mid-tibial cortical bone density, **c** total calcium, **d** ionised calcium, **e** inorganic phosphorus, and the serum 25-hydroxyvitamin D (25(OH)D) concentration. The vertical line in A and B represents observed breakpoint in 25(OH)D concentration

The breakpoint in the 25(OH)D concentration indicating a significant change in the PTH concentration occurred at 17 ng/mL (Fig. 1a). Furthermore, the serum 25(OH)D concentration was below this threshold in 37 (26.6%) rabbits. The mean PTH concentration was 6.3 pg/mL (SD ± 9.6) in rabbits with a 25(OH)D concentration below 17 ng/mL and 4.0 pg/mL (SD ± 5.9) in those with a 25(OH)D concentration 17 ng/mL or higher. The estimated effect size in the PTH concentration attributable to the breakpoint was 2.1 pg/mL (95% CI -0.7–5.l). Neither body weight nor age explained this variation.

The breakpoint for the cortical bone density was found at 19 ng/mL of 25(OH)D concentration (Fig. 1b). The mean cortical bone density was 1382 mg/cm^3^ (SD ± 132) in rabbits with a 25(OH)D concentration below 19 ng/mL and 1398 mg/cm^3^ (SD ± 65) in those with 25(OH)D concentration of 19 ng/mL or higher. The estimated effect size in the cortical density was 20 mg/cm^3^ (95% CI -65–24). Both body weight and age significantly explained this variation.

No significant breakpoints were observed between the 25(OH)D concentrations and the ionised calcium, total calcium or inorganic phosphorus concentrations (Fig. 1c–e). The mean iCa, Ca and P concentrations were similar in those rabbits with 25(OH)D concentration below 17 ng/mL and in those with 25(OH)D concentration 17 ng/mL or higher.

## Discussion

In this study of 139 pet rabbits aged 0.1 to 9.3 years, the 25-hydroxyvitamin D concentration indicating a significant change in the parathyroid hormone concentration occurred at 17 ng/mL. Furthermore, these findings suggest an almost identical breakpoint of 19 ng/mL for the mid-tibial cortical bone density, signifying a plausible level of vitamin D deficiency in pet rabbits. Accordingly, 25(OH)D concentrations below the observed breakpoint appear to associate with a higher PTH level and a lower cortical density. The breakpoint is slightly higher compared to the 12 ng/mL observed in a large human study of adults by Shah et al. (2017) [10], and similar to the 18 ng/mL observed in children aged 0.2 to 18 years by Kang et al. (2017) [9]. Yet, Sai et al. (2011) found no such breakpoint for 25(OH)D within the concentration range of 6 to 60 ng/mL [8]. Breakpoints for some bone markers (serum osteocalcin and urine N-telopeptides), however, were found at a 25(OH)D concentration of 18 ng/mL [8].

The serum 25(OH)D concentration fell below the observed PTH-related breakpoint in about 30% of the pet rabbits we studied, suggesting that every third rabbit was vitamin D deficient. The main function of vitamin D lies in maintaining the skeletal calcium balance. Decreased 24(OH)D concentration correlates therefore with increased PTH concentration which aims to maintain serum iCa, Ca and P concentrations within the normal physiological range. Although our results suggest high percentage of vitamin D deficiency among the studied pet rabbit population, biochemical parameters did not give further evidence for clinical state of secondary hyperparathyroidism in them. Shah et al. [10] reported hypocalcemia in 6.1% and hypophosphatemia in 3.4% of participants with vitamin D concentration below observed breakpoint. Their human study population comprised 11,855 participants so it is possible that our sample size lacked statistical power to reveal the presence of clinical hyperparathyroidism. Further research is needed on long-term effects of chronic vitamin D deficiency on rabbits’ bone health. Yet, vitamin D also plays an important function in many other metabolic functions. For instance, vitamin D carries anti-inflammatory and immunomodulatory effects; in humans, a deficiency is linked to several common health disorders including cardiovascular diseases, cancers, asthma, allergies and respiratory infections [14–22]. The threshold for a severe vitamin D deficiency in humans occurs at 12 ng/mL, but a minimum concentration of 30 ng/mL or even 40 ng/mL is needed for optimal cellular health [14, 16]. The vitamin D deficiency level observed in this study of pet rabbits is based on optimising bone health. Yet, further studies are needed regarding the minimum vitamin D level for optimal cellular health in pet rabbits or to determine the possible link between vitamin D deficiency and common health problems among them.

We observed no breakpoint for 25(OH)D concentrations in relation to the Ca and iCa concentrations, which agrees with results by Brommage et al. (1988) [13]. Furthermore, this reflects the fact that calcium absorption from the intestines occurs passively in rabbits and active vitamin D-dependent absorption is needed only if the dietary calcium level is low [23]. The calcium concentration in the rabbits in our study varied from 2.9 mmol/L to 4.0 mmol/L. Only two rabbits had a calcium concentration below 3.0 mmol/L, while three had levels over 3.9 mmol/L. The reference ranges of 1.51 to 4.26 mmol/L for the calcium concentration in adult rabbits (5 to 7 month depending on the size of the rabbit breed) and 1.9 to 5.3 mmol/L in juvenile (8 weeks old) rabbits were reported recently by Korn et al. (2018), without significant differences between adults and juveniles [24]. Ranges for ionised calcium were respectively 1.45 to 2.52 mmol/L and 1.2 to 1.7 mmol/L with significantly lower concentrations observed in juvenile rabbits [24]. Reported phosphate concentrations were 0.69 to 2.06 mmol/L in adults and 1.3 to 3.2 mmol/L in juvenile rabbits with significantly higher concentrations in 8 weeks old rabbits [17]. Despite the wide age range of our study, only four rabbits in our study were under 4 months old and therefore classified as juvenile and none of the medium or large sized rabbits were under 7 months old.

Accordingly, none of our rabbits were calcium deficient. The exact dietary levels of vitamin D, calcium or phosphorus among our rabbits remain unknown [5]. The adult rabbit daily calcium requirement is approximately 500 mg [25], which they typically receive by ingesting roughly 200 g of dry hay [26]. Rabbits need a lot of calcium given their continuously growing teeth, but may also receive calcium from their teeth due to continuous wear. The importance of dental wear as a source of calcium in rabbits has, however, remained unsolved. The growth rate depends on the abrasiveness of the diet [27]. Rabbits with a very abrasive diet, such as through a hay-only diet, may have more rapid dental wear and may, therefore, obtain much calcium from dental tissue.

Only one previous study has reported the parathyroid hormone concentration in 27 pet rabbits [7]. That study used a second-generation immunoradiometric assay (IRMA), which enjoyed wide use in PTH analysis among humans and in many studies of experimental rabbits [28–31]. However, manufacturing of this assay was discontinued with the introduction of third-generation immunometric assays. Since then, no papers have introduced PTH assays suitable for rabbit serum. Watson et al. (2019) measured rabbit PTH using radioimmunoassay, but neither the details of the assay nor the results related to it were published [4]. The approximately 3 years delay in PTH analysis of our samples was caused by difficulties in search of a suitable analysis for rabbit serum. The manufacturer of the presently used kit recommends using serum samples stored at -20 ºC within 2 months after sample collection. Regarding the literature, the long-term stability of PTH in serum samples varies depending on the assay [32]. Reported maximum stability for PTH in serum samples was at least 2 years in -80 ºC and 5 years in—20 ºC [32, 33]. Our samples were stored at -80 ºC.

The PTH assay kit was intended to be used for human samples. The standard material as well as the one level control was of human origin. The human control was necessary to ensure that the assay itself was performing correctly between sample batches and the laboratory work was satisfactory. The use of a control material of rabbit origin was restricted by the small volume of the samples. Our possibilities for the validation of the assay for rabbit serum was limited in dilutional parallelism and intra-assay variability. The PTH assay tubes were counted in three independent runs including standard curves. Each run included batches of control and sample tubes, which were handled at the same time (melting samples, pipetting antibody, incubating etc.). Every batch did not include standard tubes. These three runs included 17 control results used for calculating inter-assay variability. In terms of the validation parameters, the radioimmunoassay we used in this study was considered adequately suitable and valid for PTH analysis in rabbit serum.

The main challenges in our study pertain to the heterogeneity of the pet rabbit population studied and to sample collection which was not unified at same time frame in all samples to prevent effect of hormonal circadian rhythm. This heterogeneity renders interpretation more difficult compared to experimental studies of carefully chosen target groups in terms of the variability in diet and living conditions as well as breed and age. On the other hand, variation in age, breed and body sizes provide natural variation in the evaluated parameters which is useful when a novel method is assessed. The lack of adequate information on diet and living conditions is an apparent limitation of this study. The inherent variability in data, however, permits observation of low and high 25(OH)D concentrations in family-owned rabbits and results represent more the population met as patients in the veterinary clinics.

## Conclusions

The association between the parathyroid hormone and 25(OH)D concentrations changed at 17 ng/mL, marking the threshold for vitamin D deficiency in pet rabbits. The breakpoint for the mid-tibial cortical bone density at 19 ng/mL was similar to the proposed vitamin D deficiency level. We found that one-third of our rabbits had a 25(OH)D concentration below this threshold. Consequently, concerns persist regarding the high prevalence of vitamin D deficiency and its potential health consequences amongst pet rabbits.

## Methods

### Animals and study design

The Animal Experiment Board of Finland (5562/04.10.03/2011) approved this cross-sectional study of pet rabbits. Three previous studies employing the same pet rabbit cohort appear elsewhere [5, 34, 35]. In total, 174 family-owned pet rabbits (*Oryctolagus cuniculus*) participated in this study on a voluntary basis in 2012 and 2013. Amongst these, 139 (80%) rabbits were eligible for this study after clinical and radiographic examinations. Results of these examinations have been published previously [34]. We excluded rabbits with health disorders requiring veterinary treatment.

The mean age of these 139 rabbits was 2.6 years (range 0.1–9.3 years, standard deviation [SD] ± 2.0), with data missing in 5 cases; 72 (51.8%) rabbits were female (10 [13.9%] neutered) and 67 (48.2%) were male (31 [46.3%] neutered). Rabbits represented 19 different breeds, the most common of which were Dwarf Lop (*n* = 38, 27.3%) and mixed breed (*n* = 37, 26.6%).

### Data collection

For the blood sampling and bone density measurements, rabbits were sedated with a subcutaneous injection of 0.1 mg/kg medetomidine and 5 mg/kg ketamine. Blood samples taken via venipuncture from the cephalic or lateral saphenous vein were collected into tubes coated with (0.5 ml) and without anticoagulants (1 + 1 ml) as well as into a lithium-heparin syringe (0.5 ml). After blood sample collection, the plain tubes were immediately placed into an ice bath and centrifuged at a relative centrifugal force of 1485 times gravity (x g) for 10 min using refrigerated centrifuge (+ 4ºC). The serum was divided into two tubes, the first of which was frozen at -80 °C for later use and the second of which was used for biochemical analysis within 24 h after sample collection. All analyses were performed in the Central Laboratory of the Department of Equine and Small Animal Medicine in the Faculty of Veterinary Medicine at the University of Helsinki (Finland). 25(OH)D and PTH analyses were performed during autumn 2015.

The right tibia was scanned using peripheral quantitative computed tomography.^1^ For this study, the mid-diaphysis cortical bone density (CortD in mg/cm^3^) was determined as described previously [35].

After examinations, the effects of medetomidine were reversed by a subcutaneous injection of 0.25 mg/kg atipamezole.

### Biochemical and serum 25-hydroxyvitamin D analyses

The total calcium and inorganic phosphorus concentrations were determined using standard laboratory methods. The ionised calcium concentration was analysed using a standard blood-gas analyser^2^ from a heparinised syringe sample. The serum 25-hydroxyvitamin D concentrations were determined from frozen samples after melting using an enzyme immunoassay^3^ [5].

### Parathyroid hormone determination

The rabbit PTH concentration was determined from frozen serum samples after melting using a solid-phase two-site immunoradiometric assay.^4^ This assay was primarily designed for the measurement of PTH in human serum or plasma, and was used according to the manufacturer instructions with slight modifications. The reagent and sample volumes were halved given the small sample volumes available. However, the radioactivity bound to the tubes was sufficient for gamma scintillation counting.^5^ The two highest calibrators—480 and 1500 pg/mL—were excluded and two new calibrator levels—namely, a low 5.0 pg/mL and 96 pg/mL—were prepared by diluting the kit standard 50 pg/mL 1:10 and 480 pg/mL 1:5 with the zero plasma. Standards were analysed in triplicate, while samples were analysed in duplicate as recommended in three assay protocol. We report the results as the mean values. According to the standard routine, the results were calculated using six calibrator levels and four-parameter logistics fittings.

The linearity of the assay was evaluated by dilution. One rabbit sample with a high PTH concentration was serially diluted (1/2, 1/4, 1/12 and 1/36) with the zero standard of the kit (Table 3). Triplicate analyses were performed for each dilution. The observed-to-expected ratios were calculated for the dilutions. The low limit of detection (LOD) was determined from the repeated measurements of the zero standard and calculated using the mean and standard deviation (SD) of the blank measurements (mean ± 3.3 SDs). The low limit of quantification (LOQ) was set by a low PTH concentration rabbit sample with a target imprecision of approximately 5%. The PTH measurement was repeated six times and the coefficient of variation (CV%) was calculated as a percentage of the SD from the mean value of the sample. The precision of the assay was determined as the intra-assay variability from duplicate measurements of 29 rabbit samples and as an inter-assay variability from 17 measurements from the human control of the assay kit. PTH concentration range in these 29 rabbit samples was 3.5 to 49.5 pg/mL and were selected to represent all the observed PTH concentrations of the samples.

**Table 3:** Dilutional parallelism observed in rabbit serum samples for ELSA-PTH^a^ kit

Dilution	Measured (pg/mL)	Expected (pg/mL)	Measured/Expected (%)
1/2	103.4	96.1	107.6
1/4	52.7	48.1	105.6
1/ 12	16.6	16.0	103.8
1/ 36	5.4	5.3	98.2

^a^ELSA-PTH, CisBio Bioassays, Codolet, France

In the linearity assessment, the observed mean PTH concentrations were 103.4 pg/mL, 52.7 pg/mL, 16.6 pg/mL and 5.4 pg/mL, respectively, representing 117.6%, 109.7%, 103.8% and 101.9% of the expected PTH concentrations. The dilutions of the rabbit samples showed linearity over the studied range (*R*^*2*^ = 0.998). The LOD was 0.5 pg/mL. The mean for the low PTH concentration sample was 3.3 pg/mL and the variability across six measurements was 5.9%. The CV% was sufficient for setting the LOQ at 3.3 pg/mL (3.0 pg/mL given in the kit insert). The intra-assay CV% for the duplicate measurements of the rabbit plasma samples with PTH concentrations varying from 3.5 to 49.5 pg/mL was 7.7%. The inter-assay CV% of the human PTH control of the kit was 5.0% (38.1 ± 1.9 pg/mL, target value 40 ± 6 pg/mL).

### Statistical analysis

We performed first univariate correlation analyses and determined Pearson correlation coefficients (r) between 25(OH)D, PTH, iCa, Ca, and P concentrations, cortical bone density, age and body weight. Then we performed segmented linear regression analysis^6^ using the 25(OH)D concentration as the independent variable, and the iCa, Ca, P, PTH concentrations and mid-tibial cortical density as the dependent variables. In the case of a significant breakpoint detected, the values of the dependent variables above and below (including the breakpoint) were compared using the general linear model,^7^ where body weight and age served as the covariates. We set the level of significance to *P* < 0.05.

## Data Availability

The data and materials analysed during the current study are available from the corresponding author on reasonable request.

## Abbreviations

Ca: Total calcium
CI: Confidence interval
CortD: Cortical bone density
CV: Coefficient of variation
iCa: Ionised calcium
LOD: Limit of detection
LOQ: Limit of quantification
P: Phosphorus
PTH: Parathyroid hormone
SD: Standard deviation
UVB: Ultraviolet B
25(OH)D: 25-Hydroxyvitamin D

## Footnotes

1 Stratec XCT 2000 Research + , Stratec Medizintechnik GmbH, Pforzheim, Germany.

2 ABL800 Flex, Radiometer, Copenhagen, Denmark.

3 25-hydroxyvitamin D EIA kit AC-57SF1, Immonodiagnostic Systems Holdings PLC, Tyne & Wear, UK.

4 ELSA-PTH, CisBio Bioassays, Codolet, France.

5 Wallac Wizard 3" 1480 Automatic Gamma Counter, Wallac Ltd, Turku, Finland.

6 The SegReg program (www.waterlog.info/segreg.htm). Accessed Aug 28, 2020.

7 SPSS Statistics for Windows, Version 25.0. IBM Corp, Armonk, NY, USA.
